# Geographic Variation in the Diet of Opaleye (*Girella nigricans*) with Respect to Temperature and Habitat

**DOI:** 10.1371/journal.pone.0045901

**Published:** 2012-09-21

**Authors:** Michael D. Behrens, Kevin D. Lafferty

**Affiliations:** 1 Department of Ecology, Evolution and Marine Biology, University of California Santa Barbara, Santa Barbara, California, United States of America; 2 Western Ecological Research Center, United States Geological Survey, Marine Science Institute, University of California Santa Barbara, Santa Barbara, California, United States of America; Ecole Normale Supérieure de Lyon, France

## Abstract

We studied diet variation in an omnivorous fish across its range, which allowed us to test predictions about the effect of ocean temperature and habitat on herbivory. Throughout most of its geographic range, from Southern California to central Baja California, the opaleye (*Girella nigricans*) fed primarily on red and green algae, but there was significant variation in the amount of algal material in the diet among sites. The proportion of algal material in the diet was related to habitat, with algae making up a larger proportion of a fish’s diet in algal-dominated habitats than in urchin barrens. Independent of habitat, the proportion of algal material in the diet increased with environmental temperature. Analyses of stable isotopes revealed similar changes in trophic position and confirmed that these associations with diet persisted over relatively long time scales. The shift to a more herbivorous diet at warmer temperatures is in agreement with past laboratory studies on this species that show a diet-dependent change in performance with temperature and can indicate a diet shift across the species’ geographic range to meet its physiological demands. A possible plastic response to herbivory was a longer gut relative to body size. The results of this study are consistent with past findings that associate temperature with increases in the relative diversity of herbivorous fishes in tropical parts of the ocean.

## Introduction

Herbivorous fishes make up a larger proportion of overall fish diversity and abundance at tropical lower latitudes [1], [2]. Whether this is due to temperature or evolutionary history is a subject of debate [2], [3]. There are certainly exceptions to the association between herbivorous fishes and temperature. First, some herbivorous fishes live in cold water [see 3 for examples]. Second, it is not always the case that algae are sufficiently abundant at warm temperatures to support herbivores. While algal growth may increase with temperature, nutrient availability is generally lower in tropical waters and competition among grazers may reduce the standing stock of algal material [4]. Third, some fish that are nominally herbivorous may actually obtain much of their energy from detritus [3]. Although we focus on hypotheses for the general pattern of a positive association between warmer temperatures and herbivory in fishes, alternative explanations should be considered for those cases that don’t meet the pattern.

Temperature can affect ectothermic metabolism [5], diet quality [6], [7], and diet quantity [1], potentially leading to different optimal diets at different temperatures. Algae comprise a relatively low-quality food for consumers [8], and the combination of low gut passage in cold water [9] and the poor quality of algae as food might make it difficult for herbivory to supply the needs of metabolically active fishes in cold water. For other ectothermic herbivores, assuming food is unlimited, the need for high-quality food declines with temperature due to the compensatory mechanisms of increased eating and enhanced physiology [10]. As temperatures increase, fishes can digest faster [9], potentially making it possible for them to trade quality for quantity. In other words, if edible algae are sufficiently abundant and the water is sufficiently warm, herbivory could be a more energetically favorable diet strategy. In addition, the favorability of herbivory might increase with temperature if algae are relatively easier to digest at higher temperatures than at lower temperatures [4]. For instance, intestinal fermentation of algal material, used by some herbivorous fishes [8], [11], does not occur at low temperatures and its rate accelerates (initially) with temperature [1], [12].

Omnivores can be suitable study organisms for evaluating predictions of the temperature-herbivory hypothesis if their feeding habits and performance (for example, growth or digestive efficiency) can be assessed under different environmental conditions. We studied the opaleye (*Girella nigricans*), a common omnivorous member of the rocky-reef fish assemblage, in the field to better understand the potential effects of temperature on diet. The opaleye experiences a range of biotic and abiotic conditions between its latitudinal boundaries at the southern tip of Baja California and central California [13] and there is variability in feeding behavior over space and time [14]–[16]. We previously found laboratory evidence for an increase in herbivory with temperature in opaleye, where, in cold water, opaleye perform (as measured by RNA:DNA ratios) very poorly on an ad-libitum algal diet unless animal material was also available; at warmer temperature, the addition of animal material did not improve performance [17]. These data have been considered controversial support for a temperature-dependent digestive limitation on herbivory [2]. Based on these laboratory results, we predicted that opaleye would be more herbivorous at warm than cold locations due to an increase in performance at high temperatures on a purely herbivorous diet. We also suspected that variation in food availability (which varies by habitat type) could affect diet. Although omnivores employ a generalist life style, we considered that there could be some plastic responses to food availability or quality [18]. Herbivorous fishes generally have relatively longer guts than closely related carnivorous species [8]. We therefore investigated whether increased feeding on algae was associated with longer gut lengths.

Traditional studies of feeding in fishes have focused on foraging observations or stomach-content analyses [19]. Although these are important methods for determining what food items an individual organism selects, due to various degrees of digestibility among food items, observations might not accurately indicate what prey items make up most of the individual’s assimilated nutrition [20], [21]. For example, most herbivorous fishes ingest invertebrates associated with the algae they consume [8]. Analyses of stable isotope signatures (traditionally δ^13^C and δ^15^N) can help indicate trophic position because they are responsive to assimilated nutrients, and isotope signatures of consumers are typically enriched relative to their prey [22]–[25]. However, stable isotope signatures can vary over spatial and temporal scales due to different pools of carbon and nitrogen that support marine food webs [25], [26] and because primary producers use different sources of these isotopes [27], [28]. Therefore, it is important to take into account the isotopic signature of primary producers at each location when analyzing stable isotope signatures across large spatial scales [29]. The combination of traditional stomach content analysis and stable isotope signatures can be powerful because stomach content analysis determines what a fish ate during the last feeding period, whereas stable isotopes produce a signal of nutrients assimilated over months to a year [30], [31]. By using both methods, we sought to understand both short-term influences on feeding behavior in opaleye and the potential effect of those influences on assimilated nutrients over longer time periods.

In this study, we used a combination of traditional stomach-content and stable isotope analysis to investigate changes in feeding and assimilation of consumed items across a large portion of the geographic range of *G. nigricans*. The aims of this study were to (1) describe variation in the diet, (2) determine what factors (habitat, size, relative gut length, gut fullness, seasonality/date, and temperature) correlated with diet, and (3) evaluate the prediction that the proportion of algal material in the diet would increase with environmental temperature as predicted by the laboratory findings on the effect of temperature and diet on the energetics of this species [17].

## Materials and Methods

### Sample Collection

**Figure 1:**
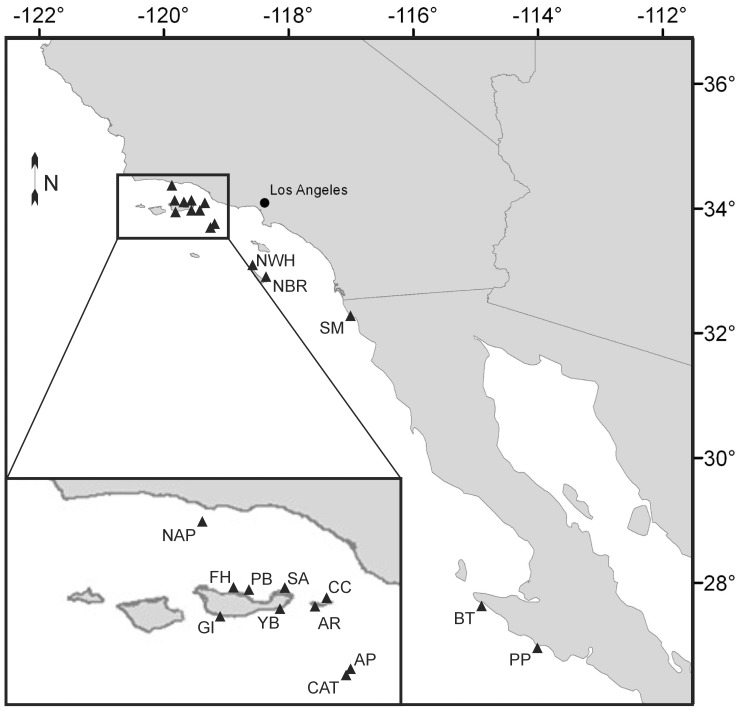
Fish collection locations in Southern California and Baja California. See Table 1 for habitat designations.

*Girella nigricans* were collected by spear from shallow-water (3–15 m) rocky reefs throughout the Channel Islands and the mainland of Southern California and Northern Baja California between June 2001 and Sept. 2003 on an opportunistic basis during the spring and summer (Fig. 1 and Table 1). All fish were collected in the afternoon (1200–1800 hours) to control for variation in feeding throughout the day and increase the volume of food in the stomach. We collected 4–11 fish from each of the 15 locations (Table 1), after which we removed white muscle samples and the digestive tracts from each fish. After dissection, the white axial muscle samples and digestive tracts were frozen at −20°C. For each fish, the standard length (SL) and the length of the digestive tract (GL) from the esophagus to the anus were measured. Also, at each site, samples of 3–5 algal and/or seagrass species on which we observed *G. nigricans* feeding were collected for stable isotope analysis. These samples primarily consisted of red algal taxa that were also commonly observed in the stomachs of this species. Algal samples were washed in freshwater to remove invertebrates.

**Table 1:** Fish collection sites, habitat, temperature, sample size, and fish size range (Standard length – SL in mm.) and percent dry mass (±1 standard error) of various dietary categories found in fish stomachs.

Site	Hab	Collection Temp.	Annual SST	N	Collection Date	SL	% Algae	Red Algae	Green Algae	Brown Algae	Seagrass	Crustacea	Mollusca	Hydroid	Bryozoa	Annelida	Echinoderm
NAP	B	13	15.5	4	16-Aug-01	285±16	45.36±26.35	35.21±20.33	25.27±13.97	–	–	1.78±1.32	–	36.85±31.72	–	0.54±0.29	0.35±0.35
FH	B	18.6	15.6	9	12-Sep-01	263±19	86.36±5.62	70.19±9.61	14.16±4.85	2.01±2.01	–	6.13±2.54	0.83±0.48	5.84±3.62	0.7±0.40	0.13±0.13	–
SA	B	19.2	16	8	10-Sep-01	180±15	53.24±13.60	29.87±9.06	23.38±11.93	–	–	25.62±11.50	–	10.01±6.14	–	11.13±7.13	–
PB	B	18.9	15.7	11	12-Sep-01	267±13	83.04±6.95	69.55±12.23	7.1±5.37	0.08±0.08	6.31±6.31	9.83±6.45	-	1.22±0.39	5.74±3.86	0.11±0.09	0.05±0.05
CC	A	19.1	16.1	9	13-Sep-01	268±11	79.33±8.75	25.75±7.20	44.23±9.68	–	9.35±8.72	13.74±8.13	0.28±0.22	3.84±2.68	2.81±2.72	–	–
AR	A	19.4	16.1	10	13-Sep-01	253±6	92.06±1.59	90.99±1.41	1.08±0.71	–	–	2.7±1.13	0.05±0.05	5.16±1.83	–	0.01±0.01	0.01±0.01
YB	A	15.5	16.1	8	10-Sep-01	275±7	83.55±6.09	37.49±12.90	46±14.67	–	0.07±0.05	1.95±1.11	–	12.93±4.86	1.56±1.56	–	–
GI	A	16.3	15.7	10	11-Sep-01	253±16	79.87±8.54	95.84±0.67	0.37±0.37	0.66±0.66	–	1.22±0.48	0.12±0.12	0.91±0.19	-	0.09±0.02	0.79±0.79
AP	A	14.7	16.7	6	11-Jun-01	246±6	98.53±0.57	80.94±3.88	13.54±4.61	–	4.05±4.05	0.44±0.13	0.38±0.38	0.63±0.22	-	–	0.01±0.01
CAT	B	16.4	16.7	9	13-Jun-01	254±4	67.58±10.17	21.59±5.93	35.41±12.65	10.58±10.58	–	2.58±1.42	-	29.23±10.02	0.61±0.61	–	–
NWH	A	15.9	17.4	6	2-Jun-02	217±17	59.8±16.91	39.41±25.42	39.19±29.38	2.1±1.54	–	15.94±15.83	1.16±1.16	2.19±0.72	–	–	–
NBR	A	14.8	17.2	10	31-May-02	255±8	93.98±5.79	50.83±9.08	26.11±8.16	–	17.04±6.23	0.13±0.05	0.03±0.03	5.86±5.78	–	–	–
SM	A	19	17.2	6	7-Sep-03	174±10	95.12±4.13	95.12±4.13	–	–	–	0.62±0.24	4.24±4.23	0.01±0.01	–	–	–
BT	A	20	18.6	10	10-Sep-03	121±6	98.82±0.80	98.77±0.81	–	–	–	0.63±0.49	0.12±0.06	0.12±0.12	–	0.37±0.33	–
PP	A	21	19.5	6	11-Sep-03	125±14	97.58±0.85	97.47±0.82	–	–	–	1.87±0.77	0.18±0.09	0.32±0.32	–	0.09±0.06	0.07±0.07

Habitat (Hab): A-Algal Dominated; B-urchin barrens. Sites sorted by latitude from North to South (Fig. 1).

### Diet Analysis

To address how the diet of *G. nigricans* varied among locations, the amount of algal material in the diet was determined using the dry mass of animal and algal material in the stomach of each fish. After thawing the digestive tracts, all materials were washed from the stomach and sorted into gross taxonomic groupings (Table 1). All dietary items from each sample were dried at 70°C for at least 48 hours and then weighed. The dry mass of the animal and algal taxa from the stomach were determined separately and used to calculate the percent contribution to the diet of each taxa and the percent algal material in the stomach of each fish. Non-dietary items (e.g. small rocks) were removed and excluded from the dietary analysis.

Analysis of Variance (ANOVA) was conducted to test for significant variation in the percent algal material in the diet of *G. nigricans* among sites. Then, to determine what factors were associated with feeding in *G. nigricans*, a general linear model (GLM) was developed to test the independent effects of habitat, standard length, collection day (day of year), total stomach contents (dry mass (g)), and water temperature on the day of collection (range across locations = 13.0–21.0°C) on the percent algal material in the stomach. Habitats varied among sites (see Table 1) and were classified as either algal dominated (including kelp forests and sites with high abundance of benthic algae), or urchin barrens. To assess the relationship between digestive morphology and diet, a partial correlation analysis was used to test for an association between diet and relative gut length, after the removal of the effect of standard length. Proportion data were arcsine square-root transformed to meet the assumptions of the statistical models. All statistical analyses were conducted using JMP [32].

### Stable Isotopes

The algae and the white muscle samples from all fish were thawed. All encrusting organisms were removed from the surface of the algae with a scalpel. Samples were dried at 70°C for at least 48 hours and then ground to a fine powder using a mortar and pestle. The samples were analyzed for nitrogen stable isotope signatures at the Marine Science Institute Analytical Laboratory (University of California, Santa Barbara) using a continuous-flow isotope ratio mass spectrometer (Finnigan Delta Plus Advantage). The results were reported in δ^15^N values in the standard per mil units relative to international standards (atmospheric N_2_). Our analysis was limited to nitrogen stable isotopes due to the larger average fractionation between tropic levels as compared to carbon stable isotopes [22], [23].

ANOVA was conducted to test for significant variation in δ^15^N values for *G. nigricans* among sites. There was a significant association between mean δ^15^N values of fish and algae among sites (r = 0.863, F_1,13_ = 37.890, p<0.001), indicating that the δ^15^N values are likely influenced by local pools of nitrogen. Therefore, a standardized measure was produced that would approximate the relative trophic position of each fish above the algae at a given location. As a standardized measure, the trophic enrichment between the isotopic signature (δ^15^N) of each individual fish and the mean algal isotope signature at a given site were determined and this relative trophic position was used in further analyses.

To determine what factors affected isotopic signatures in *G. nigricans*, a GLM was used to test the independent effects of habitat (as described above), standard length, collection day (day of year), and mean sea surface temperature (SST) during the 12 months preceding collection (range across locations = 15.5–19.5°C) on relative trophic position. Annual SSTs for each location were used, rather than temperature on the day of collection as in the diet analysis, because stable isotope methods integrate feeding patterns over a much longer period than do stomach content analysis [30], [31]. To assess the relationship between digestive morphology and relative trophic level, a partial correlation analysis was used to test for an association between relative trophic position and relative gut length, after the removal of the effect of standard length.

### Temperature Data

Temperature data were obtained for the date of collection and 12 months preceding collections from various sources for use in further analyses. These different time scales were used to better match the time scales over which feeding was investigated. Temperature data for the date of collection were subtidal water temperatures from temperature recorders. Long-term data from temperature recorders were not available for all of our fish collection locations, so sea surface temperatures (SST) from weekly day-and-night composite Pathfinder 5 AVHRR satellite four km sea surface temperature images were used to produce annual mean temperatures (NOAA National Oceanographic Data Center: http://www.nodc.noaa.gov). These annual means were calculated by averaging the three pixel by three pixel square centered on each site (for an effective resolution of 12 km). While this level of resolution is likely too coarse to discriminate between some of our northern sites (Figure 1), the decision to use these data was made to present a consistent data type across all sites.

### Ethics Statement

All necessary permits were obtained for the described field studies. Permission to conduct research in Channel Islands National Park was obtained from the Park Superintendent. For all other locations, no specific permissions were required for these locations/activities. These locations were not privately-owned or protected. The field studies did not involve endangered or protected species. This study was carried out with approval of the Institutional Animal Care and Use Committee at the University of California, Santa Barbara (protocol # 617).

## Results

Across all sites, opaleye were omnivorous, consuming, on average, 82% algae and seagrasses. However, fish at 25% of the sites consumed >90% algae and seagrasses, indicating they were primarily herbivorous at many sites. Opaleye selectively foraged on red and green algae, as shown by the relative lack of brown algae in the guts of fish from algal-dominated sites (including kelp forests). This is not surprising because not all readily available algae (e.g. kelps) are digestible [20], [21] and this likely shapes diet preference. Crustaceans and hydroids made up most of the animal material in the diet (Table 1). The amount of algal material in the gut contents showed a high degree of variation among sites, with values ranging from 45 to 99% algae (Table 1, ANOVA, F_14,107_ = 3.912, p<0.001). Habitat types did not differ in temperature on the day of collection (t_126_ = 0.417, p = 0.681), but did differ in annual mean temperature (t_126_ = 5.674, p<0.0001), with algal dominated sites being warmer than barrens. For this reason, we included habitat in models with temperature to better estimate the independent effects of temperature and habitat and associated differences in food availability. Trophic position of *G. nigricans* (relative to algae) also varied among sites (F_14,113_ = 8.417, p<0.0001). Level of herbivory (% algae in the diet) was only weakly negatively correlated with the relative trophic position (r = −0.174, F_1,120_ = 3.760, p = 0.055), suggesting that diet on the day of collection was only somewhat representative of the feeding history of a fish and that high levels of variation exist between the two measures. The variation in fish diet and relative trophic position was investigated further to determine which factors were associated with this variation.

### Factors Associated with % Algae in the Diet

**Figure 2:**
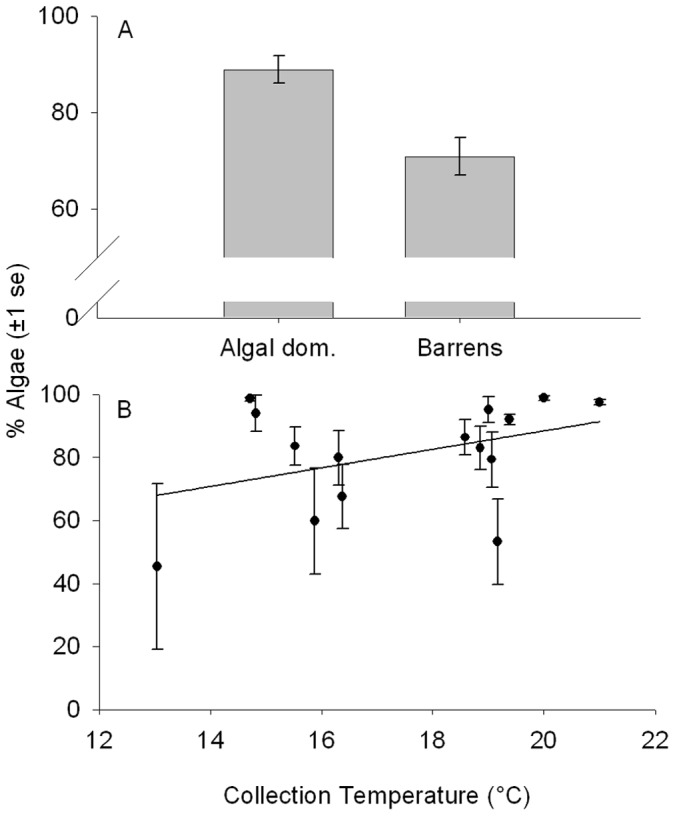
Associations between diet of *G. nigricans* and habitat and temperature. A**-**Mean percent algae in the diet for each habitat type. B-Mean percent algae in the diet for each location plotted against temperature on the day of collection (°C). Error bars ±1 standard error.

**Figure 3:**
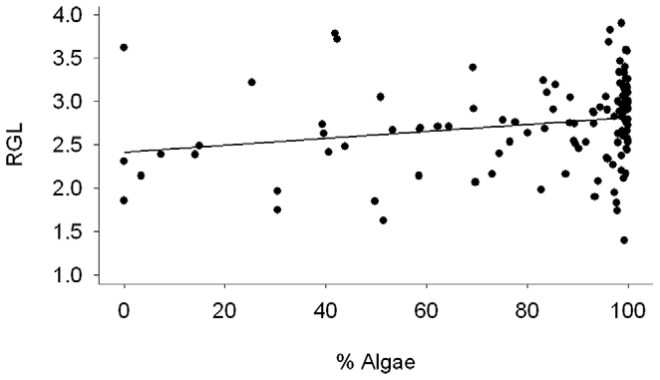
Associations between relative gut length and diet of *G. nigricans*.

The diet of *G. nigricans* varied among sites and was associated with various environmental and morphological factors, including habitat, and temperature, following removal of the effects of the other variables. The fraction of algal material was significantly associated with habitat (Fig. 2; GLM; R^2^ = 0.100, F_1,116_ = 12.694, p<0.001). *G. nigricans* showed a greater fraction of algae in their diet in algal-dominated habitats than in urchin barrens. The percentage of the diet consisting of algae increased with environmental temperature (Fig. 2; GLM; R^2^ = 0.040, F_1,116_ = 4.412, p = 0.038). There was not a significant association between the fraction of algal material and standard length (GLM; F_1,116_ = 0.0003, p = 0.985), collection day (GLM; F_1,116_ = 1.436, p = 0.233), or total stomach contents (GLM; F_1,116_ = 0.986, p = 0.322). Relative gut length was positively correlated with the percent algae in the diet (Fig. 3; r_p_ = 0.215, t_117_ = 2.432, p = 0.016) when controlling for fish length.

### Factors Associated with Stable Isotopes Relative Trophic Position

**Figure 4:**
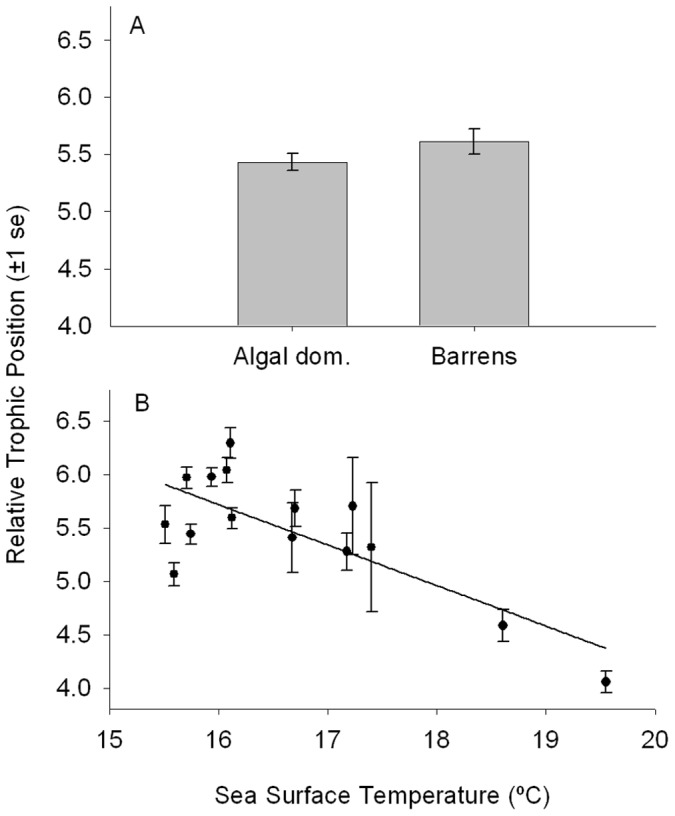
Associations between stable isotope signatures, habitat and temperature. A-mean relative trophic position (difference in fish δ^15^N from location algal mean δ^15^N) for each habitat type. B**-**mean relative trophic position for each location plotted against annual mean sea surface temperature (°C). Error bars ±1 standard error.

Environmental and morphological factors were associated with the relative trophic position of a fish. The relative trophic position (difference in δ^15^N values from algae) in *G. nigricans* was not associated with fish length (GLM; R^2^ = 0.008, F_1,120_ = 1.035, p = 0.311) or the date of collection (GLM; R^2^ = 0.005, F_1,120_ = 0.637, p = 0.426), but was significantly associated with habitat (Fig. 4; GLM; R^2^ = 0.043, F_1,120_ = 5.375 p = 0.022). The relative trophic position was lower in algal-dominated habitats than in urchin barrens. The relative trophic position declined with increasing temperature (Fig. 4; GLM; R^2^ = 0.207, F_1,124_ = 31.305, p<0.0001). Relative trophic position was not correlated with relative gut length (r_p_ = −0.0961, t_122_ = −1.066, p = 0.289) when controlling for fish length.

## Discussion

Herbivory in opaleye was greater where algae were common and increased with water temperature. The temperature effect occurred at two temporal scales. Opaleye had more algae in their guts when it was warm and the trophic position of opaleye was lower where long-term temperatures were higher. Opaleye appeared to respond to a more herbivorous diet by increasing allocation to gut length. These results suggest that food availability and digestive physiology affect diet in this omnivorous fish. These results are consistent with experimental findings that suggest herbivory is more profitable at warm temperatures than cold temperatures [17]. These finding may help explain why herbivory appears to be more common at lower latitudes.

Short term (% algal material in the gut) and long term (relative trophic position) indices of herbivory varied significantly among sites, but were only weakly associated with one another. This lack of association between stomach contents and stable isotopes is not rare (see [33], [34] for examples). These differences seen in our two approaches might be due to the different temporal scales of the analyses and variation in the digestibility of various items consumed by opaleye. Stomach contents likely reflect food consumed in the habitat of capture. However, habitat state at many of the collection locations is known to vary over time [35] and it is likely that fish feed in many different habitats over the time that the isotopic signature is produced. Additionally, this is probably why habitat of capture was not as strong a predictor of long-term diet as it was for short-term diet (see below). Relative trophic position, as estimated by stable isotope analysis, is driven by which dietary items are digested and assimilated into an organism’s tissues and not all dietary items are equally digestible [20], [21]. Therefore, dietary items that are the most readily digested will have a disproportionate impact on stable isotope ratios, potentially leading to differences between stomach contents and stable isotope ratios.

We were surprised that our measures of herbivory did not increase with fish size because past studies had indicated that omnivores can shift toward herbivory as adults [36]–[38]. Specifically, it has been reported that small, intertidal opaleye were carnivorous and subtidal adults were herbivorous, suggesting an ontogenetic diet shift at 50 mm SL [13], [39]. However, such studies did not control for environmental difference in the habitats of adult and juvenile fishes, so it is possible that what appeared to be an ontogenetic shift was actually a habitat effect. Our other data indicate that small size is not a constraint on herbivory as opaleye as small as 36 mm can be primarily herbivorous [12]. We were also surprised that herbivory was not associated with fuller guts because one response to the low digestibility and quality of an herbivorous diet should be to increase the amount of food consumed [40], [41].

Habitat affected short- and long-term measures of herbivory in opaleye in ways that suggested algal availability increased herbivory. Algae were more common in the guts of fish from sites where algae were abundant and relative trophic position was lowest at algal dominated sites. For most species of algae, abundance is higher in kelp forests and sites with dominant benthic algal assemblages than in urchin barrens; in contrast, habitat or community state less predictably affects invertebrate abundance [35], [42], [43]. However, the proportion of variation in both diet and relative trophic level explained by habitat alone were low, 10% and 4% respectively. The qualitative habitat classifications used in this study were extremely course and likely limited our ability to better detect the effect of habitat and food availability on feeding. Associations between levels of herbivory and quantitative measures of the availability of edible algae would be a more direct test of the hypothesis that algal availability alters herbivory in this omnivore. However, even with these course descriptions that likely do not capture the variation within each habitat type, the effect of habitat on diet was highly significant.

Neither diet nor the relative trophic position varied with the date of sample collection. This lack of change over time is somewhat surprising, because related Kyphosids in the subfamily Girellinae have been shown to have seasonal diet shifts [44]. However, it should be noted that all samples were collected from late spring to late summer, potentially masking longer-scale seasonal patterns. Additionally, if seasonal changes are due to variation in temperature, covariation between date of sample collection and temperature might limit our ability to detect seasonal shifts in diet. The use of annual SST to analyze relative trophic position likely limits covariation between date of sample collection and temperature; however, changes in relative trophic position across the sampling period were still not detected, supporting the finding of a lack of seasonality.

The association between temperature and herbivory is correlational and could be driven by other factors we did not measure. In particular, the relative abundance of algal-associated invertebrates could have been higher at cold sites though we note that opaleye do not perform well on an herbivorous diet in cold water [17]. Spatial autocorrelations among herbivory, habitat and temperature are another alternative explanation because algal-dominated sites were more common in the Southern portion of the sampling area. However, the correlation between habitat and temperature only existed with mean annual SST, therefore this spatial autocorrelation should not affect diet analyses.

There is some circumstantial evidence that other omnivorous fishes can actively adjust their diet according to temperature. Blue rockfish (*Sebastes mystinus*) consume significantly more algae during warm-water periods, though this is possibly due to limited abundance of preferred foods [45]. Pinfish (*Lagodon rhomboids*) consume a greater proportion of animal material in cold water than warmer water, and refuse to consume algae below 17°C (M. Hay unpubl.). Additionally, seawater temperature can affect diet choice in cold-water invertebrates, leading to selective consumption of higher quality diets at cold temperatures [5].

The relationship between temperature and diet or trophic position was consistent over multiple time scales, as suggested by stomach-content and stable isotope analyses, indicating that this association translates from feeding behavior to assimilation of nutrients via digestive processes. Although the temperature explained a relatively low proportion of variation in gut contents (4%), it explained far more of the stable isotope data (20%). Therefore, while temperature may only slightly modify feeding behavior, it may have a greater impact on digestive processes, which influence stable isotope signatures. Laboratory studies in this species predict a transition from herbivory to omnivory with a decline in water temperature [17]. Even though the association between temperature and diet was relatively small, this should not be too surprising. Actual feeding behavior is likely driven by a range of factors, including feeding preference, prey availability, evolutionary constraints on morphology and other factors that we were unable to measure.

In addition to changing diet according to temperature, opaleye appear to have some relatively rapid physiological responses to herbivory. Herbivores tend to have longer guts to facilitate digestion of plant and algal material [8]. Changes in gut length in opaleye might have reflected plastic responses in digestive morphology to the consumption of various foods, as seen in Cichlid fishes [18]. The ability to digest low-quality foods, such as algae, increases with gut length, favoring herbivory in older, larger fish [8], [36]. In general, relative gut length increases with size in herbivorous fishes and this change accompanies ontogenetic diet shifts [8], [36], [37]. Even in carnivorous fish families, those species that exhibit occasional or seasonal herbivory show concomitant increases in relative gut length [45], [46]. There was an association between digestive morphology and diet, but not relative trophic position. This difference in results is potentially due to the different time scales of these measurements. Laboratory studies of herbivorous Stichaeid fishes indicate that relative gut length can change is as few eight weeks (M. Behrens unpubl.). Therefore, changes in diet may accompany changes in relative gut length before they impact the stable isotope signatures of the organism.

In conclusion, feeding and sources of assimilated nutrients in *Girella nigricans* appear to be associated with many factors, including habitat, digestive system morphology, and water temperature. Both stomach content and stable isotope analyses suggest this species consumes and assimilates a greater proportion of plant matter as temperature increases, which confirms our prediction, based on laboratory studies in this species, and adds support to the temperature-herbivory hypothesis to potentially explain the distribution of herbivorous fishes [1], [2]. Additionally, if these findings are consistent across many fish species, then temperature increases due to climate change may lead to increased levels of herbivory in marine systems potentially affecting entire systems as predicted by recent studies [47].
